# PET/MRI for staging patients with Hodgkin lymphoma: equivalent results with PET/CT in a prospective trial

**DOI:** 10.1007/s00277-021-04537-5

**Published:** 2021-04-28

**Authors:** M. Picardi, C. Cavaliere, R. Della Pepa, E. Nicolai, A. Soricelli, C. Giordano, N. Pugliese, M.G. Rascato, I. Cappuccio, G. Campagna, C. Cerchione, E. Vigliar, G. Troncone, M. Mascolo, M. Franzese, R. Castaldo, M. Salvatore, F. Pane

**Affiliations:** 1 Department of Clinical Medicine and Surgery, Federico II University Medical School, Via Sergio Pansini, 5, 80131 Naples, Italy; 2 Department of Nuclear Medicine and Radiology, IRCCS SDN, Via Emanuele Gianturco 113, 80143 Naples, Italy; 3 Department of Radiology, University of Naples Parthenope -IRCCS SDN, Via Ferdinando Acton 38, 80143 Naples, Italy; 4 Department of Public Health, Federico II University Medical School Naples, Via Sergio Pansini, 5, 80131 Naples, Italy; 5 Department of Advanced Biomedical Sciences, Federico II University Medical School Naples, Via Sergio Pansini, 5, 80131 Naples, Italy

**Keywords:** PET/MRI, Hodgkin lymphoma, PET/CT

## Abstract

To compare FDG-PET/unenhanced MRI and FDG-PET/diagnostic CT in detecting infiltration in patients with newly diagnosed Hodgkin lymphoma (HL). The endpoint was equivalence between PET/MRI and PET/CT in correctly defining the revised Ann Arbor staging system. Seventy consecutive patients with classical-HL were prospectively investigated for nodal and extra-nodal involvement during pretreatment staging with same-day PET/CT and PET/MRI. Findings indicative of malignancy with the imaging procedures were regarded as lymphoma infiltration; in case of discrepancy, positive-biopsy and/or response to treatment were evidenced as lymphoma. Sixty of the 70 (86%) patients were evaluable having completed the staging program. Disease staging based on either PET/MRI or PET/CT was correct for 54 of the 60 patients (90% *vs.* 90%), with difference between proportions of 0.0 (95% CI, −9 to 9%; *P*=0.034 for the equivalence test). As compared with reference standard, invasion of lymph nodes was identified with PET/MRI in 100% and with PET/CT in 100%, of the spleen with PET/MRI in 66% and PET/CT in 55%, of the lung with PET/MRI in 60% and PET/CT in 100%, of the liver with PET/MRI in 67% and PET/CT in 100%, and of the bone with PET/MRI in 100% and PET/CT in 50%. The only statistically significant difference between PET/MRI and PET/CT was observed in bony infiltration detection rates. For PET/CT, iodinate contrast medium infusions’ average was 86 mL, and exposure to ionizing radiation was estimated to be 4-fold higher than PET/MRI. PET/MRI is a promising safe new alternative in the care of patients with HL.

**Supplementary Information:**

The online version contains supplementary material available at 10.1007/s00277-021-04537-5.

## Introduction

Hodgkin lymphoma (HL) requires accurate staging to plana proper therapy (1). According to several international guidelines, whole-body ^18^F-fluoro-deoxy-glucose (FDG)-positron emission tomography (PET) together with computed tomography (CT) is fundamental for this purpose (FDG-PET/CT) (1, 2). The Lugano Classification recommends a full diagnostic CT, by using intravenous (i.v.) injected iodinate contrast medium and current at full dose (the so-called FDG-PET/diagnostic CT), as part of hybrid imaging for improving nodal measurements and extra-nodal disease detection especially in clinical trials (1–4). However, concerns have been raised over the safety profile of such diagnostic tool, especially when new generation PET/CT scanners are not employed (5–7). Because of the increased survival of patients with HL, the current strategy is to maximize cure rates while minimizing toxicity (1). In line with this paradigm, it is desirable to avoid side effects induced by iodinate contrast medium and ionizing radiation dose exposure of the CT part of a PET/CT examination (5–7).

With advances in technology, whole-body magnetic resonance imaging (MRI) is now technically feasible. Hybrid PET with MRI systems for scanning the entire body have been approved by the US Food and Drug Administration for clinical use, providing functional and morphological information with excellent soft tissue contrast (PET/MRI) (8). However, exact data on the diagnostic accuracy of PET/MRI compared with PET/CT in patients with FDG-avid lymphomas are missing. Studies on this issue are methodologically flawed for several reasons (9, 10): different subtypes of lymphoma are included; patients are scanned at different time points (i.e., at staging, response assessment and sometimes surveillance); numerous patients have not any detectable disease at the time of the scan; very few extra-nodal localizations; devices with post hoc fused PET and MRI images; few appropriate sequences of MR images resulting in differences between PET/MRI and PET/CT in anatomic localization of focal lesions; finally, selected acquisitions of MRI with scanning time generally longer than with the PET/CT (making the examination unlikely to be tolerated by the elderly or unfit patients) (8). As proved in preliminary studies for oncological disease, the definition of valid protocols is a key requisite for correct PET/MR images. For breast cancer staging, high-quality PET/MRI examination provided results comparable to PET/CT not only in terms of malignant lesion classification (8) but also more accurate in terms of extra-nodal disease identification, particularly in the case of occult bony metastases (11). The impact of PET/MRI with new generation equipment, with specific anatomic MRI sequences simultaneously acquired with PET, in staging FDG-avid lymphoma of a homogeneous series of patients has not been well determined to date (8–10).

The aim of this trial was to compare the staging evaluation obtained with FDG-PET/unenhanced MRI (experimental imaging approach) with that obtained with FDG-PET/diagnostic CT (conventional imaging approach) in a cohort of patients with newly diagnosed HL (*clinicaltrials.gov*: registration no. NCT03042247). To the best of our knowledge, this is the first study prospectively assessing the performance of a dedicated PET/MRI protocol in a large series of patients characterized by the same lymphoma sub-type and time point of imaging evaluation. Moreover, multiple nodal and extra-nodal sites of disease were analyzed in the study, allowing a broad evaluation of lesion detection by the different imaging modalities.

## Materials and methods

### Participants

The study was conducted in the Hematology Unit of the Federico II University of Naples, and the Department of Nuclear Medicine and Radiology of the IRCCS SDN of Naples, (Italy). All necessary approvals were obtained from the ethics committees, and a specific consent form for imaging procedures was obtained from each patient according to the Declaration of Helsinki.

From January 2017 to June 2019, consecutive patients with HL referred to the Hematology Unit of the Federico II University of Naples for FDG-PET-based pretreatment staging according to the revised Ann Arbor system of the Lugano Classification (3, 4) were screened for this study. The imaging scans were performed at the Department of Nuclear Medicine and Radiology of the IRCCS SDN of Naples.

Inclusion criteria were as follows: a histopathologic diagnosis of classical HL (12–14), age 18 to 70 years, no previous anti-lymphomatous treatment, and induction chemotherapy planned with intent-to-cure (1).

### Study design

This prospective trial was designed to primarily compare the accuracy of FDG-PET/unenhanced MRI with that of FDG-PET/full dose contrast-enhanced CT in staging patients with untreated HL. As secondary endpoint, we calculated the magnitude of i.v. iodinate contrast medium and ionizing radiation dose exposure reduction obtained with FDG-PET/MRI as compared with the FDG-PET/CT.

In the study, we placed emphasis on the sensitivity of the experimental and conventional imaging approaches in detecting malignant lesions. Sensitivity for PET/MRI and PET/CT in identifying lymphoma involvement was calculated as the number of correct classifications divided by those positive for malignancy according to the reference standard. The results of a combined imaging modality (i.e., PET/MRI or PET/CT) were defined correct (true-positive or true-negative) if in agreement with the results of the other combined imaging modality (concordant study). If malignancy was detected with only one combined imaging modality (discordance: e.g., positive lesion in the skeletal on PET/MRI, while no clear malignant findings at PET/CT, or vice versa), the reference standard was taken into account for the final interpretation and for establishing which imaging modality correctly defined staging.

### Reference standard

Pathology (by surgical intervention or core-needle biopsy) (13, 14) served as primary standard of reference for lymphomatous involvement detection. In the case of non-availability of pathology, an integration of follow-up investigations with routine clinical assessments, laboratory, and imaging (including gray-scale ultrasonography, contrast-enhanced ultrasonography, MRI, diagnostic CT, and/or FDG-PET) lasting at least 3 months post-chemotherapy regimen served as the secondary standard of reference. In particular, lesions were correctly characterized as lymphomatous if they had decreased size and/or decreased FDG uptake, or increased size and/or increased FDG uptake after chemotherapy.

### Staging work-up

Staging was performed by using the conventional procedures practiced at our institution (Supplemental Appendix 1) (15–17). In addition, patients underwent whole-body FDG-PET/MRI. The treatment was planned only on the basis of conventional staging results.

### Scanning workflows

As specifically required by the Minister of Health of Italy and by the institutional review board (IRB), PET/CT was always performed first followed by PET/MRI. FDG-PET/MRI was performed only in patients undergoing same-day FDG-PET/CT with the same radiotracer injection and radioactivity dose as required for a standalone PET/CT, at the Department of Nuclear Medicine and Radiology of the IRCCS SDN of Naples. Thus, PET/CT and PET/MRI were performed sequentially in the same patient on the same day. A single workflow was used.

### PET/CT protocol

Patients underwent, on a single occasion, whole-body FDG-PET/contrast-enhanced CT with a combined in-line system (Discovery 710; GE Medical Systems, Milwaukee, WI) that integrates a four-detector-row spiral CT with a PET scanner (17). The relevant technical details are reported in Supplemental Appendix Table 1 and Supplemental Appendix 2.

### PET/MRI protocol

Soon after PET/CT completion, the patient underwent a second examination with the other combined imaging modality. PET/MRI was performed with a 3T hybrid scanner (mMRBiograph, Siemens Healthcare, Erlangen, Germany) equipped with three 32 channels body coil, to cover the thorax, abdomen, and pelvis areas, and 12 channels phased array brain. These coils were combined into a multichannel whole-body coil by using total imaging matrix technology. For PET/MRI scans, no new FDG was injected; no MRI contrast agents were administered. The relevant technical details are reported in Supplemental Appendix Table 2 and Supplemental Appendix 3.

### Image interpretation

For FDG-PET (visual assessment for interpreting scans), CT, and MRI studies, accepted published imaging criteria were used to evaluate for lymphoma involvement (3, 4, 8). To give a solid basis for noninvasive diagnosing of lymphoma involvement, in particular to avoid false-positive results, we perceived that the malignant findings should be confirmed by both imaging techniques for each combined modality, i.e., FDG focal uptake at PET scans and positive-CT or positive-MRI. Thus, it was needed a concordance between metabolic and anatomic data to finally diagnosing lymphoma, according to the international guidelines (3, 4).

Then, the readers recorded for each patient the disease stage according to the revised Ann Arbor system, based on each combined imaging modality (Supplemental Appendix 4).

### Radiation dosimetry

Radiation exposure from the FDG-acquisition portions of PET studies was calculated using standard millisievert (mSv) conversion factors from publication 80 of the International Commission on Radiological Protection (18). Radiation exposure from the CT portion of PET/diagnostic CT study was estimated using the method outlined in report 96 of the American Association of Physicists in Medicine (19).

### Treatment strategy

All patients underwent a chemotherapy regimen used for HL, named ABVD (Doxorubicin, Bleomycin, Vinblastine, and Dacarbazine) (1). Each course was repeated every 4 weeks according to the following schedule: 25 mg/m^2^ doxorubicin administered intravenously on day 1 and day 15, 10 mg/m^2^ bleomycin administered intravenously on day 1 and day 15, 6 mg/m^2^ vinblastine administered intravenously on day 1 and day 15, and 375 mg/m^2^ dacarbazine administered intravenously on day 1 and day 15. Patients with limited disease (stages I–II) received from two to four courses of ABVD followed by involved-field irradiation (20–30 Gy). The number of ABVD courses as well as the irradiation doses was determined by the presence of unfavorable prognostic factors (1). Patients with advanced disease (stages III–IV) received six courses of ABVD followed by residual mass irradiation (32 Gy), if any (1, 20).

For the study purpose, the lymphomatous lesions detected at pretreatment staging were re-evaluated at the end of chemotherapy in order to monitor lesion size (long axis diameter) and/or FDG uptake.

### Statistical analysis

Our sample size calculation was based on the primary efficacy endpoint, i.e., equivalence between PET/MRI and PET/CT in correctly defining revised Ann Arbor staging (3, 4).

The analysis of the power of the study was performed with supposition based on the scientific literature that the sensitivity in detecting lymphoma infiltration at the conventional imaging approach was ≥95% (1–4), whereas the sensitivity in detecting lymphoma infiltration was about 92% with the experimental imaging approach (8–10). Therefore, in our model, the maximum tolerable difference between conventional imaging and experimental imaging sensitivity was supposed to be 10%. Thus, a 95% confidence interval (CI) for the difference in sensitivity rates within 10% was required to consider equivalent the two imaging approaches. The null hypothesis was *D*=(PET/MR_sensitivity_ − PET/CT_sensitivity_) >10%, where *D* is the difference in sensitivity between the two imaging approaches and the alternative hypothesis was *D=*(PET/MR_sensitivity_ − PET/CT_sensitivity_)≤10%. We compared relative differences between proportions by computing a two-sided 95% CI for the difference in proportions to claim equivalence (21). Assuming a 15% loss of patients in the efficacy analysis calculation, a sample of 60 patients who could be evaluated in each group was required for a two-sided type I error of 5% and a power of 80%(*α* error= 0.05, *β* error= 0.20, effect size= 0.55).

Statistical analysis was performed by using specific software (R version 3.6.0). The Cohen’s kappa statistic (*κ*), Pearson’s chi-squared tests, and Fisher’s exact test were used for statistical evaluations (Supplemental Appendix 5).

This trial is registered with ClinicalTrials.gov, number NCT03042247.

## Results

### Patient demographics

Between 2 January 2017 and 31 July 2019, a total of 70 consecutive patients with newly diagnosed and untreated classical HL were scheduled to undergo on the same-day FDG-PET/diagnostic CT first, followed by FDG-PET/unenhanced MRI for pretreatment staging. Of them, 10/70 (14%) patients failed MRI examination with consent withdrawal and thus were excluded. The remaining 60 patients well tolerated the whole scanning workflows of PET/CT and PET/MRI studies and were included in the final assessment.

**Fig. 1:**
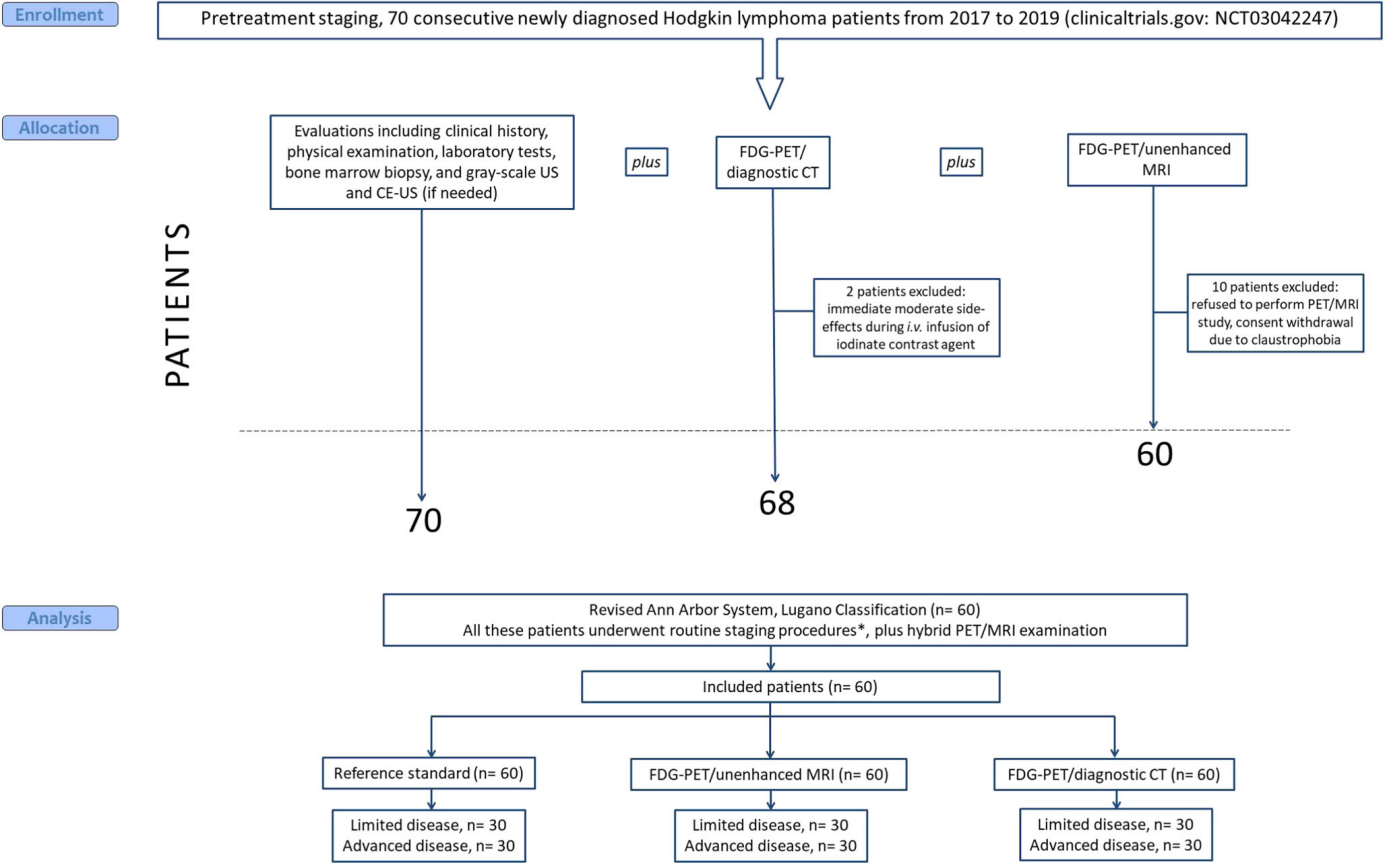
Consort diagram. PET/MRI and PET/CT enable equivalent determination of the tumor stage. FDG-PET, ^18^F-fluoro-deoxy-glucose positron emission tomography; CT, computed tomography; MRI, magnetic resonance imaging; US, ultrasonography; CE-US, contrast-enhanced ultrasonography; i.v., intravenous. *Routine staging procedures: see supplemental Appendix 1

The complete staging work-up consisted in routine assessments [clinical and laboratory evaluations together with bone marrow biopsy, gray-scale ultrasonography, and contrast-enhanced ultrasonography (if needed)], and FDG-PET/diagnostic CT scans and experimental investigation with PET/MRI. The entire study flow is shown in Fig. 1.

Table 1 shows the clinical and histopathology characteristics of 60 analyzed patients. The median age was 40 years with a range of 18–70 years. Females were 52% of patients. B symptoms were recorded in 61% of patients. Erythrocyte sedimentation rate was ≥50 mm in 58% of patients. Nodular sclerosis sub-type of classical HL was present in 68% of patients. Bone marrow biopsy was positive for HL infiltration in 10% of patients.

**Table 1:** Clinical characteristics of the study population

Variable	Prospective cohort of patients undergoing pretreatment staging with conventional and experimental imaging approaches: baseline features
No. of patients	60
Median age (range), years	40 (18–70)
Sex	
Male	29 (48%)
Female	31 (52%)
Hodgkin lymphoma subtypes (who classification)*	
Nodular sclerosis	41 (68%)
Mixed cellularity	14 (23%)
Lymphocyte rich	3 (5%)
Lymphocyte depleted	2 (4%)
B symptoms	
Fever	32 (53%)
Sweats	21 (35%)
Weight loss ≥10%	16 (26%)
Erythrocyte sedimentation rate ≥50 mm	35 (58%)
Spleen invasion at CE-US	9 (15%)
Bone marrow infiltration at biopsy	6 (10%)

Unless otherwise indicated, data are the number of patients

*CE-US* contrast-enhanced ultrasonography

*WHO* World Health Organization

*Biopsy-proven Hodgkin lymphoma. Histologic samples were obtained by ultrasonography-guided core-needle cutting biopsy in 40 patients and surgical excisional biopsy in 20 patients

### Revised Ann Arbor stage

**Fig. 2:**
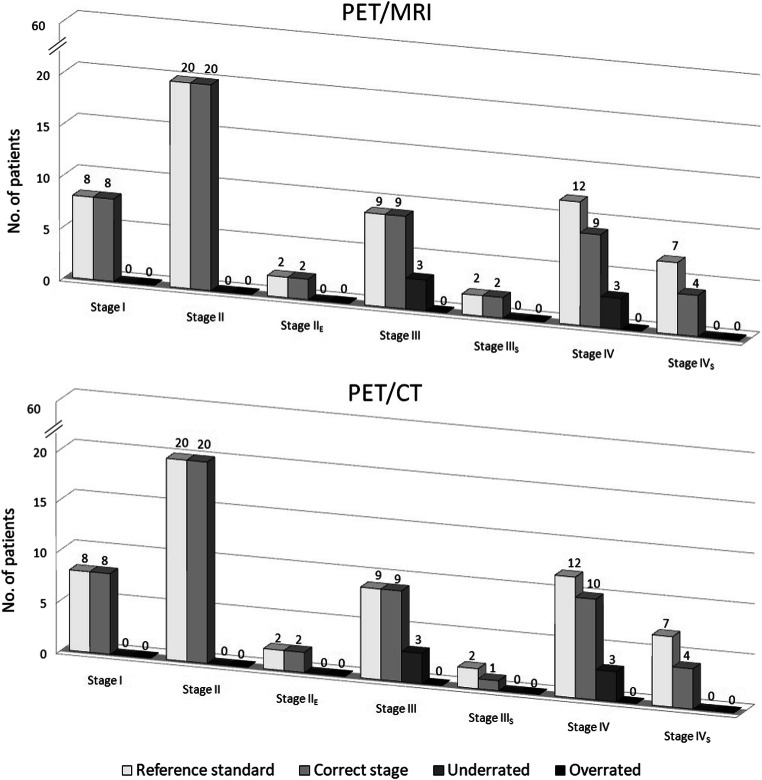
Revised Ann Arbor staging according to the Lugano Classification. Based on the reference standard, final disease stage was I for 8 patients (14%), II for 22 patients (37%; II_E_ for 2 of them), III for 11 patients (18%; III_s_ for 2 of them), and IV for 19 patients (31%; IV_s_ for 7 of them). No overrated stage was observed, owing to the stringent imaging criteria for malignant findings, i.e., positive-PET combined with positive-CT and/or positive-MRI. PET, ^18^F-fluoro-deoxy-glucose positron emission tomography; MRI, unenhanced magnetic resonance imaging; CT, full-dose contrast enhanced computed tomography

There was excellent concordance of correct classification of positive findings between the two combined imaging modalities. Among the 549 [nodal (*n*= 492), extra-nodal (*n*= 39), and splenic (*n*= 18)] malignant lesions detected on the reference standard, 520 (94.7%) were classified the same way by reference standard as they were by PET/MRI and PET/CT, representing almost perfect agreement [*κ*= 0.93 (95% CI, 0.90–0.95); *P*<0.001; by Cohen’s test]. Consequently, PET/MRI correctly staged disease in 54 of the 60 patients as PET/CT. The accuracy rate of staging status was 90% for both PET/MRI approach and PET/CT approach (difference between proportions, 0.0; 95% CI, −0.09–0.09; *P*= .034, for the equivalence test). In addition, there was almost perfect agreement in staging between the two combined imaging modalities, with a kappa value of 0.85 (95% CI, 0.72–0.98; *P*< 0.001, by Cohen’s test). The rated tumor stages in different imaging approach datasets including reference standard are given in Fig. 2.

### Changes in staging

By comparing the experimental imaging approach versus conventional imaging approach, PET/MRI correctly upstaged 5% of patients (*n*= 3) and incorrectly downstaged 5% of patients (*n*= 3). In addition, 5% of patients (*n*= 3) were concordantly mis-staged by PET/MRI and PET/CT. The remaining patients were correctly and concordantly staged by both combined imaging modalities (Fig. 2).

The three patients, who were correctly assigned to a higher stage by PET/MRI, only marginally changed their status. They remained in the extensive disease group, moving from an originally stage III (according to PET/CT scans) to IV with bone involvement (*n*= 2 cases) and to III_s_ with nodes above and under the diaphragm and spleen involvement (*n*= 1 case).

In the three patients incorrectly assigned to a lower stage by PET/MRI, the experimental imaging approach did not see nodular infiltration by disease in the liver (*n*= 2 cases) and lung (*n*= 1 case), erroneously allocating the patients in stage III instead of stage IV.

In the three cases incorrectly underrated by PET/CT and PET/MRI, both imaging diagnostic tools did not see spleen invasion by lymphoma: according to the reference standard, the patients moved from an originally stage IV (as defined by both combined imaging modalities) to IV_s_.

### Lesion classification discordance

Table 2 shows discordant PET/MRI and/or PET/CT classifications for 29 lesions which were depicted as malignant according to the reference standard. Of these, 15 (52%) lesions were misclassified by PET/CT, eight (28%) lesions were concordantly misclassified by PET/MRI and PET/CT, and six (20%) lesions were misclassified by PET/MRI.

**Table 2:** Discordant PET/MRI and/or PET/CT findings compared with reference standard in classifying 29 lesions

Lesion location	No. of lesions	Lesion size in cm, median (range)	Lesion detected on morphological imaging		Standardized uptake value of FDG-PET SUV_max_		Lesion classification		
			MRI	CT	PET/MRI	PET/CT	PET/MRI	PET/CT	Reference standard*
					SUV_max_, median (range)	SUV_max_, median (range)			
Lumbar vertebrae	5	0.8 (0.5–1.0)	Yes	No	4.1 (3.9–5.4)	3.9 (3.0–4.0)	Malignant	Benign	Malignant
Pelvic bones	3	1.0 (0.5–1.2)	Yes	No	4.0 (3.5–5.4)	3.8 (3.0–4.0)	Malignant	Benign	Malignant
Ribs	2	0.8 (0.5–1.0)	Yes	No	3.9 (3.8–4.0)	3.6 (2.9–4.5)	Malignant	Benign	Malignant
Spleen	5	1.5 (1.2–1.8)	Yes	Yes	4.9 (3.8–5.8)	2.5 (2.4–2.9)	Malignant	Benign	Malignant
Spleen	8	0.8 (0.5–1.0)	No	No	1.5 (1.0–1.8)	1.0 (1.0–1.8)	Benign	Benign	Malignant
Liver	4	1.0 (1.0–1.5)	No	Yes	3.9 (3.4–4.4)	4.0 (3.5–4.6)	Benign	Malignant	Malignant
Lung	2	2.0 (2.0–2.0)	No	Yes	3.5 (3.0–4.0)	3.7 (3.5–4.0)	Benign	Malignant	Malignant

*PET/MRI*, ^18^F-fluoro-deoxy-glucose positron emission tomography/unenhanced magnetic resonance imaging; *PET/CT*, FDG-PET/full-dose contrast-enhanced computed tomography; *Suv*_*max*_, maximal standardized uptake value; *see text (“Materials and methods” section)

**Fig. 3:**
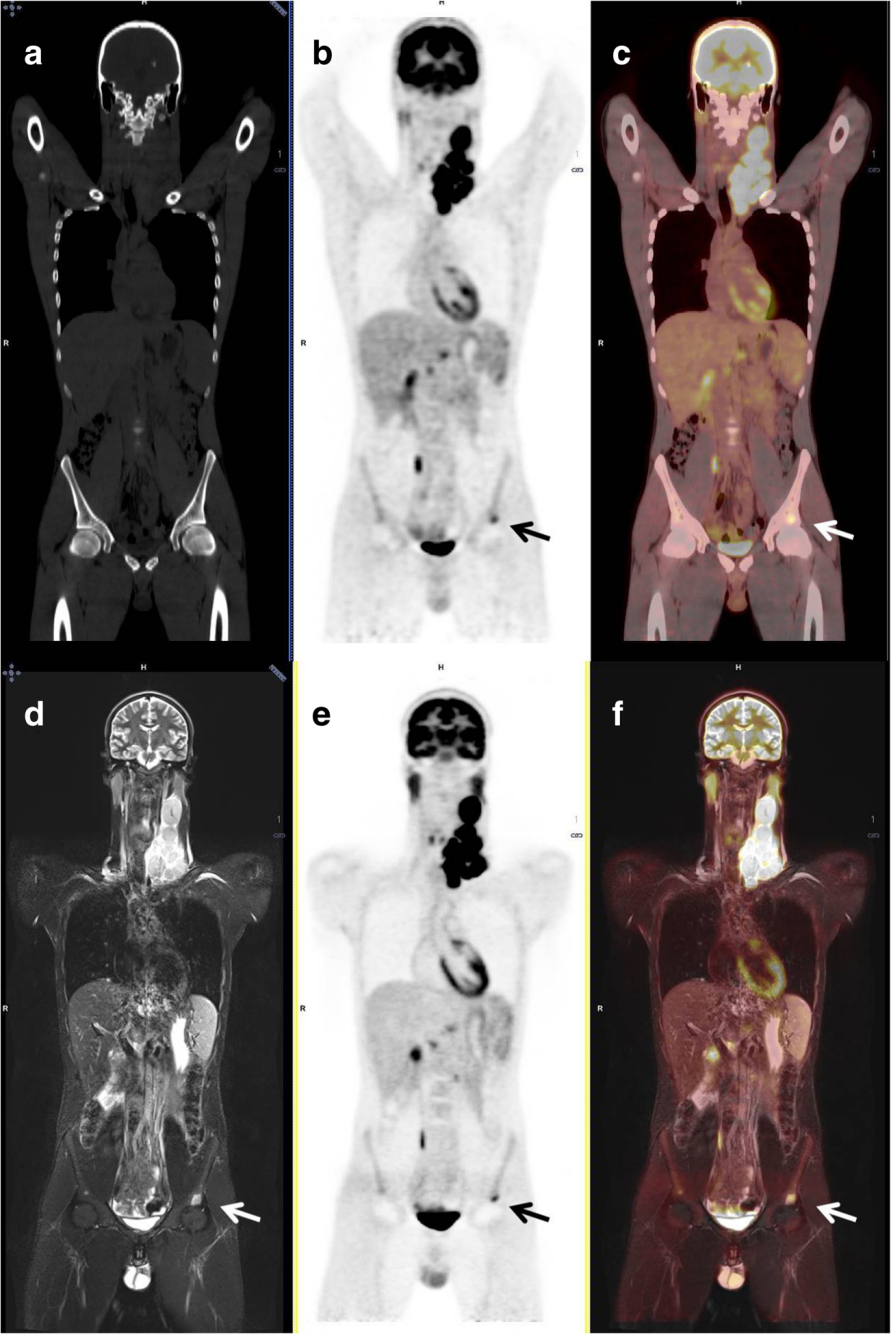
Coronal CT (**a**), coronal PET from PET/CT (**b**), fused coronal PET/CT (**c**), coronal STIR (**d**), coronal PET from PET/MRI (**e**), and fused coronal PET/MRI (**f**). FDG-avid left iliac bony lymphomatous lesion (above acetabulum; arrows) can be observed in the PET images obtained from both the PET/CT and the PET/MRI scans. However, no anatomic correlate is visible on CT, whereas it is clearly visible in the STIR image (see Supplemental Appendix Table 2 and Supplemental Appendix 3)

Ten skeletal lesions were incorrectly characterized as nonmalignant on PET/CT, whereas the same lesions were properly characterized as malignant on PET/MRI. This was the result of the combined information from FDG uptake and the entire setting of MRI sequences of our protocol, particularly useful for bony site assessment. MRI provided an anatomic correlate for FDG-avid lesions without a definite corresponding CT alteration. The lesions were seated at lumbar vertebrae (*n*= 5), pelvic bones (*n*= 3), and ribs (*n*= 2), and had median size of 0.8 cm (range, 0.5–1.2). They presented with FDG activity slightly above mediastinal blood pool levels [median SUV_max_ of 4.0 (range, 3.5–5.4)] at co-registered PET (Fig. 3). For radiological details, see Supplemental Appendix Table 2 and Supplemental Appendix 3 and 4.

Five >1.1-cm nodular lesions (median size, 1.5 cm; range, 1.2–1.8 cm) in the spleen were incorrectly classified as benign inflammatory process on PET/CT compared with proper malignant classification on PET/MRI. This discordant classification was due to differences in the interpretation of the increased FDG uptake of nodules during PET/MRI study (performed later) as compared with PET/CT study (performed earlier): median SUV_max_ of 4.9 (range, 3.8–5.8) on PET/MRI compared with median SUV_max_ of 2.5 (range, 2.4–2.9) on PET/CT.

The cause of eight lesion concordant misclassifications by using PET/MRI and PET/CT was a difference in perceived anatomic localization of <1-cm nodular lesions in splenic parenchyma, as compared with the reference standard (see Supplemental Appendix 4).

Finally, six nodular lesions (liver, 4; and lung, 2) with FDG focal uptakes were correctly identified as malignant on PET/CT, whereas the FDG uptakes were erroneously believed to be physiologic accumulations in the gut (*n*= 4), or in normal lung tissue (*n*= 2) on PET/MRI (Table 2).

### Image validation

For the 29 discordant lesions above reported, verification by histology was available for three pelvic bones lesions (bone biopsy) and two hepatic lesions (ultrasound-guided core-needle cutting biopsy). The remaining lesions were validated by follow-up imaging scans comprising contrast-enhanced ultrasonography (for 13 lesions in the spleen), FDG-PET and MRI (for 7 lesions in the skeleton), and FDG-PET/diagnostic CT (for 2 lesions in the lung, and 2 lesions in the liver).

### Results at lymph node and extra-nodal sites

For imaging details of nodal and extra-nodal findings, see Supplemental Appendix 4.

### Nonionic iodinate contrast medium infusion and radiation dosimetry

The average of i.v. nonionic iodinate contrast medium employed for the diagnostic CT of PET/CT scans is shown in Table 3.

**Table 3:** Ionizing radiation dose exposure and intravenous infusion of iodinate contrast agent related to FDG-PET/CT and FDG-PET/MRI studies

Modality	Effective ionizing radiation dose (mSv)		Percentage of total dose(Mean ± SD)
	Mean ± SD	Range	
^18^F-FDG-PET Low-dose CT Full-dose CT MRI	5.2 ± 1.5 2.8 ± 1.0 11.9 ± 3.6 NA	3.3–5.8 1.8–3.8 8.8–16.2 NA	26 ± 9.8 14 ± 10 60 ± 12 NA
	Infusion of iodinate contrast agent (mL)		
	Mean ± SD	Range	
Contrast-enhanced CT MRI	85.88 ± 9.2 NA	80–100 NA	100% NA

*FDG-PET*, ^18^F-fluoro-deoxy-glucose positron emission tomography; *low-dose CT*, computed tomography for attenuation correction; *full-dose CT*, computed tomography for diagnostic purpose; *MRI*, magnetic resonance imaging; *mSv*, millisievert; *NA*, not applicable; *SD*, standard deviation

Mean effective dose of ionizing radiation exposure for FDG-PET/diagnostic CT scans amounted to 19.9 mSv (range, 13.9–25.8), with FDG-PET accounting for 5.2±1.5 mSv (26.1%; Table 3).

### Treatment and follow-up

Thirty patients with limited disease received from two (stages I, 8 cases) to four (stages II, 22 cases) courses of ABVD followed by involved-field irradiation. The remaining patients with advanced stages received six courses of ABVD followed by residual mass irradiation in 18 cases.

The median follow-up was 19 months (range 3.0–32 months).

## Discussion

In this study, we showed that PET/MRI was able to distinguish the sites involved by disease with similar accuracy as PET/diagnostic CT (the conventional frontline imaging diagnostic approach) (1, 2). With the experimental imaging approach, we identified only a minority (5%) of patients necessarily requiring PET/diagnostic CT for correctly mapping disease. Importantly, we proved that most patients (90%) were perfectly staged by PET/MRI. The remaining 5% of patients was concordantly mis-staged by both combined imaging modalities. Therefore, the study objective to show equivalence between PET/MRI and PET/diagnostic CT in properly defining disease stage was achieved, being the 95 percent confidence interval for the difference in staging rates between experimental and conventional imaging approaches within 10 percentage points in either direction (the pre-specified equivalence margin for the study endpoint). Accordingly, PET/MRI and PET/diagnostic CT did play the same critical role in affecting baseline therapeutic decisions and management in our population, as patients were staged as limited disease (*n*= 30) and advanced disease (*n*= 30) by reference standard and by both PET/CT and PET/MRI (Fig. 1) (1, 2).

For the secondary endpoint in this trial, the comparison was significantly disadvantageous for PET/diagnostic CT. Compared with FDG-PET/unenhanced MRI, staging with FDG-PET/full dose contrast-enhanced CT systematically carried nonionic iodinate medium infusions (average dose needed per patient with FDG-PET/diagnostic CT was 86 mL) and considerably higher amounts of ionizing radiation exposure (estimated dose needed per patient with FDG-PET/diagnostic CT was about 4-fold higher). These features consisted of three main points we believe to be important (5–7). First, the incidence of nonionic iodinate contrast-induced renal damage has been estimated to be 3% in patients without risk factors but can rise to 30% among patients with concomitant exposure to nephrotoxic drugs such as cytotoxic agents or immunosuppressants (5). Contrast medium-related acute kidney injury is the third most common cause of hospital-acquired acute renal failure with major consequences for patients (e.g., dialysis) and increased mortality (5). Second, iodinate contrast i.v. injection can cause extra-renal toxicity too. In a review of the tolerance of contrast medium administration in 1514 patients (6), Lapi et al. reported immediate (nausea, vomiting, headache, hypotension, bronchospasm, glottal obstruction, local or generalized urticaria, palpebral edema, rash, and itching) or delayed (skin abnormality, diarrhea, fever, myalgia, arthralgia, and abdominal pain) adverse events (all of mild or moderate grade) in 178 (11.3%) patients. Finally, increased exposure to ionizing irradiation from medical imaging can also be a concern (7). Fabritius et al. reported on a series of 55 consecutive patients over the first year after HL diagnosis (22). The authors showed that diagnostic CT of the chest, abdomen, and pelvis was the largest contributor (responsible for more than 90%) to irradiation from medical imaging, with an average effective-dose delivered for each patient of 16 mSv and an excess life-time attributable risk to develop secondary cancer (including leukemia or solid tumors) per 100 patients of about one.

**Fig. 4:**
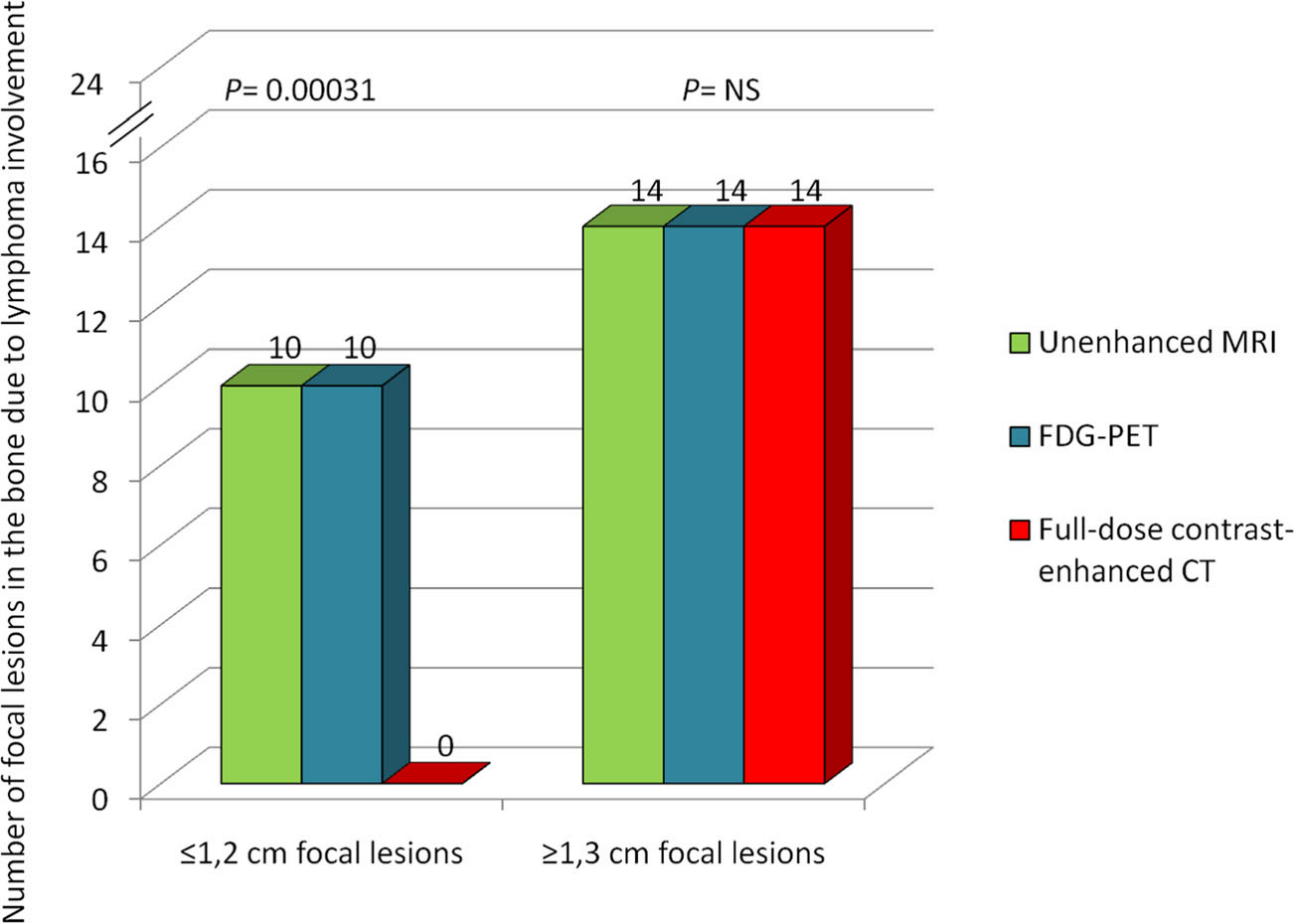
Graph depicts the diagnostic sensitivity of each imaging technique in detecting bone focal lesions involved by lymphoma according to lesion size (long axis). NS, not significant; MRI, magnetic resonance imaging; FDG-PET, ^18^F-fluoro-deoxy-glucose positron emission tomography; CT, computed tomography

According to several international guidelines, a bone marrow biopsy is no longer indicated at staging in patients with HL undergoing PET/CT evaluation, given the high sensitivity of PET/CT for detecting bony involvement (1–4). Noteworthy, we demonstrated that PET/whole-body MRI was significantly more sensitive in identifying lesions positive for lymphoma in the skeleton than was PET/CT. Thus, if a bone marrow biopsy-sparing staging diagnostic work-up is required, FDG-PET/whole body MRI can excellently replace FDG-PET/CT. In our study, PET/MRI showed in the bone invasion by lymphoma in more patients [10/60 (16.6%) on PET/MRI vs*.* 5/60 (8.3%) on PET/CT; *P*= 0.01, by Pearson’s chi-squared test] and overall more lymphomatous lesions (24 on PET/MRI vs*.* 14 on PET/CT; *P*= 0.0004, by Pearson’s chi-squared test) than PET/CT. Among the 14 bony lesions greater than 1.2 cm occurring in five patients, all were correctly classified as malignant with both PET/MRI and PET/CT. By contrast, among the 10 bony lesions 1.2 cm or smaller occurring in the remaining five patients, PET/MRI identified lymphoma involvement in all 10 lesions and diagnostic CT reported normal appearance of skeleton in all five patients (Fig. 4). In those patients with skeletal lesions with positive PET/MRI and negative PET/CT, osseous lymphoma involvement was proved through the pathological reference standard, i.e., bone biopsy, in three cases and follow-up imaging scans in the remaining two cases.

Our study suffers from several major limitations. First, this trial was conducted in one single center. PET/MRI constitutes an innovative technique for which experience and consensus regarding imaging protocols is lacking. Therefore, studies from other institutions are needed to largely assess inter-observer and inter-equipment imaging variability (8). Second, PET/diagnostic CT proved to be better to identify FDG-avid infiltration of the liver and lung than PET/unenhanced MRI, likely due to the i.v. infusion of nonionic iodinate contrast agent during CT examinations (2–4). Another reason for the sparse sensitivity of PET/MRI particularly within the lungs was incorrect tissue segmentation by PET/MRI attenuation-correction algorithm, leading to a soft-tissue lesion misclassification as normal parenchyma (8). Third, an additional limitation might have been related to the delta time between PET/CT (acquired ~60 min after FDG injection) and PET/MRI (acquired ~100 min after FDG injection), due to legal and IRB requirements (Supplemental Appendix Tables 1 and 2) (23–25). Delayed PET acquisitions might demonstrate lower background activity and improved lesion visibility (8, 24, 25). Fourth, PET/MRI was not performed in 14% of scheduled patients for low compliance. The imaging time of about 60 min following PET/CT study likely degraded PET/MRI scans in this minority sub-group of patients. Our PET/MRI protocol included several sequences aimed at comprehensive whole-body staging, at the cost of a moderate fast examination. We are in the process of protocol refinement reducing the duration of PET/MRI study. Fifth, most neo-generation PET/CT scanners can acquire a much better contrast-enhanced CT than what was included in this study, in terms of higher diagnostic quality and especially lower ionizing radiation dose protocol. Finally, set-up costs are likely higher for PET/MRI than PET/CT. Thus, PET/MRI could remain a limited resource in most countries for economic reasons (8).

Nonetheless, despite limitations, some important conclusions can be drawn. In our series, hybrid PET/MRI equipment with dedicated program proved to be especially useful, synergistically increasing the overall diagnostic yield of whole-body exams. The results of our trial add to the existing body of literature by providing new evidence that PET/MRI performs just as PET/diagnostic CT in staging patients with HL (8). Importantly, the use of PET/MRI in lieu of PET/CT allows to escape nonionic iodinate contrast medium and reduce ionizing radiation exposure (5–7, 22). Therefore, FDG-PET/unenhanced MRI could be an alternative to FDG-PET/full-dose contrast-enhanced CT as frontline imaging diagnostic tool when i.v. contrast medium-induced renal and extra-renal injury, and secondary cancer-risk due to increased ionizing radiation dose need to be avoided.

### **Author contribution**

M.P. and F.P. designed the research; F.P., M.P., C.C., R.D.P., C.G., and N.P. performed the research and wrote the paper; A.S., E.N., M.G.R., I.C., G.C., C.Ce., E.V., G.T., M.M., M.F., R.C., and M.S. collected data; N.P. analyzed data; F.P. and M.P. performed the final revision of the manuscript.

## Supplementary Information

ESM 1 (DOCX 42 kb)
