# Supplementary material for: PET/MRI for staging patients with Hodgkin lymphoma: equivalent results with PET/CT in a prospective trial
Source: Ann Hematol. 2021 Apr 28;100(6):1525–35. doi: 10.1007/s00277-021-04537-5 (PMC8116299; doi:10.1007/s00277-021-04537-5)
Supplement: Supplementary file 1 — (DOCX 42 kb) [file 277_2021_4537_MOESM1_ESM.docx]

**Supplemental Appendix, 1:**

Staging was performed by using the conventional procedures practiced atour institution—that is, clinical history, physical examination, laboratory tests, gray-scale ultrasonography, power-Doppler ultrasonography, contrast-enhanced ultrasonography, whole-body FDG-PET/diagnostic CT, and bone marrow biopsy.(15-17) The integration of these procedures led to mapping disease sites, according to the revised Ann Arbor system of the Lugano Classification.(3, 4) In addition, for the study purpose, patients underwent whole-body FDG-PET/MRI. Since FDG-PET/MRI was used for experimental purposes, the treatment was planned only on the basis of the results of conventional staging.

Exclusion criteria from the study were unwillingness to participate in the study, contraindications to iodinate contrast agent injection,(5, 6) pregnancy, claustrophobia, MRI-incompatible medical devices (e.g., cardiac pacemakers, neurostimulators, cochlear implants, and insulin pumps), or possible presence of metallic fragments in the body.

**Supplemental Appendix,2**:

Patients underwent, on a single occasion, whole-body FDG-PET/contrast-enhanced CT with a combined in-line system (Discovery 710; GE Medical Systems, Milwaukee, WI) that integrates a four-detector-row spiral CT with a PET scanner, as described.(17) FDG was produced in on-site cyclotron and chemistry facilities. All patients (fasted for at least 8 hours before the studies) had a blood glucose level of less than 150 mg/dL before FDG injection. A dose of 5.0 ± 1 megabecquerel (MBq)/kg of FDG was *i.v.* injected before imaging. PET scans were carried out from the vertex to upper thigh, with 3D emission scans of 4 min per bed position. The ordered subset expectation maximization algorithm was applied to ratio sinograms using attenuation-weighted iterative reconstruction (three iterations, 18 subsets) and subsequent smoothing with a Hanning ﬁlter. Comparable axial resolutions (full width at half maximum 4.25 mm) were obtained according to the National Electrical Manufacturers Association NU 2001 test procedure. Unenhanced low-dose CT using dose modulation and pitch of 0.938 for segmented attenuation correction was carried out in each patient. Immediately after, PET scans were acquired covering the same ﬁeld of view as the CT. Images were acquired over the field of view at 120 seconds per bed position. The maximal standardized uptake value (SUV_max_) was also evaluated. Then, contrast-enhanced CT was carried out using current at a maximum of 380 milliampere second (mAs) and *i.v.* infusion of contrast medium.

The diagnostic CT part of PET/CT examination was performed with the *i.v.* infusion of iodinate contrast agent, lopamidol (Iopamiro 370; Bracco Imaging, Milan, Italy), using a power injector at a rate of 2 ml/sec with a dose of 80 ml in patients weighing <80 kg, and a dose of 100 ml in patients weighing >80 kg. Bolus care function was used to acquire diagnostic quality arterial phase images of the upper abdomen, portal venous phase images of the whole body and delayed phase of the abdomen and pelvis.

**Supplemental Appendix,3**:

Soon after PET/CT completion, the patient was transferred to the second combined imaging modality where a second examination was performed. PET/MRI was performed with a 3T hybrid scanner (mMRBiograph, Siemens Healthcare, Erlangen, Germany) equipped with three 32 channels body coil, to cover the thorax, abdomen and pelvis areas, and 12 channels phased array brain. These coils were combined into a multichannel whole-body coil by using total imaging matrix technology. PET/MRI scans began without new FDG injection; no MRI contrast agents were administered. For each bed position,a T1-weighted Dixon gradient-echo sequence was acquired in the coronal 3D volume interpolated breath-hold examination (VIBE) of approximately 20 seconds. This sequence was acquired first for attenuation correction. It was used to generate an attenuation map with an MRI-based segmentation method that separated water, fat, soft-tissue, lung, and background attenuations. Immediately after, the following MRI sequences were performed in the trans-axial plane simultaneously with PET through multiple stations while the patient was breathing freely: a coronal short tau inversion recovery (STIR), axial diffusion-weighted imaging (DWI) with b values of 50 and 800 s/mm² and reconstruction of apparent diffusion coefficient (ADC) maps, and axial and coronal T2 Half Fourier Acquisition Single Shot Turbo Spin Echo (HASTE). A coronal T1-weighted fat satured VIBE image was acquired after PET completion.

Images post-processing were archived using the IDS7 image archiving and communication system (Sectra, Linkoping, Sweden). PET/CT image post-processing was performed using a specific workstation (GE AW Volume Share 5 Workstation). Post-processing and analyses of PET/MRI images were done using a Syngovia workstation (Siemens Healthcare).

**Supplemental Appendix, 4:**

PET/CT and PET/MRI were executed and read by board-certified expert radiologists and nuclear medicine physicians (AS, EN, CC and MS who have more than 10 years expertise with PET, CT and MRI) in consensus. Each hybrid study was evaluated as a whole. When assessing the PET/CT images, the readers simultaneously checked the CT, PET and co-registered PET/CT images. If the readers were assessing the PET/MRI images, they simultaneously evaluated the sequences reported above. The readers could modify the window settings as needed for each combined imaging modality. Specifically, the readers searched for the occurrence, number, size (the maximum diameter) and location of lymphomatous lesions on a site-specific analysis. Nodal groups included Waldeyer ring, right and left cervical, right and left axillary, right and left internal mammary or diaphragmatic, anterior mediastinal or paratracheal, right and left hilar, subcarinalor posterior mediastinal, celiac or superior mesenteric, hepatic and splenic hilar, retroperitoneal,inferior mesenteric, right and left iliac and right and left inguinal regions. In addition,several extra nodal regions were analyzed, including lungs, liver, and bone, as well as spleen and other different organs and tissues.

*Results at lymph node sites*

PET/MRI identified in 60 patients 492 lymphomatous lymphadenopathies: median number of lymph nodes per patient was 6 with a range of 1-27, median of long axis diameters was 4 cm with a range of 2-12 cm, and involved sites were at neck (n= 208), axillary (n= 98), mediastinum (n= 76) and abdominal-pelvis (n= 110). PET/CT identified in 60 patients 492 lymphomatous lymphadenopathies. The agreement between PET/CT and PET/MRI for number, size and location of pathological lymphadenopathies in each patient was perfect with k values of 1, 1 and 1 respectively (by Cohen’s test).

The reference standard classified as malignant 492 lymph nodes in 60 patients, who were available for analysis. Compared with the reference standard, there was no difference between PET/MRI and PET/CT for sensitivity in detecting lymph node involvement by lymphoma (100% *vs.* 100%, respectively; *P*= 1, by Pearson’s chi-squared test).

*Results at spleen sites*

No patient had unequivocal palpable splenomegaly.

PET/MRI identified in the spleen of six patients ten lymphomatous nodules: median number of nodules per patient was 1 with a range of 1-3, median of long axis diameters was 1.5 cm with a range of 1.2-3.0 cm, and involved sites were at upper (n= 3), middle (n= 3) and lower (n= 4) third of the spleen. PET/CT identified in the spleen of five patients five lymphomatous nodules. The agreement between PET/CT and PET/MRI for number, size and location of pathological nodules in the spleen was from moderate to substantial with k values of 0.61, 0.43 and 0.64 respectively (by Cohen’s test).

The reference standard classified as malignant 18 nodules in the spleen of nine patients, who were available for analysis. Compared with the reference standard, there was no significant difference between PET/MRI and PET/CT for sensitivity in detecting splenic invasion by lymphoma (67% *vs.* 56%, respectively; *P*= 0.63, by Pearson’s chi-squared test).

Unexpectedly, three out of nine patients (33%) with nodules with positive-reference standard for lymphoma infiltration in the spleen had negative PET, CT and MRI findings. FDG-PET, full dose contrast-enhanced CT and unenhanced MRI resulted in a high number of false-negative examinations in detecting splenic invasion by lymphoma and this was particularly true for nodules measuring 1 cm or less. Despite contiguous sections, there is a risk that CT and/or MRI scanning might not effectively depict small neoplastic nodules, due to partial volume effect, thus impairing the success of CT or MRI assessment. The interface with the lung for the spleen lesions is another reason for MRI failure. In addition, small nodules may have an insufficient number of neoplastic cells to show increased FDG uptake compared with the background activity of the bordering parenchyma, thus impairing the success of PET assessment. Contrast-enhanced ultrasonography is more sensitive in identifying small nodules positive for lymphoma in the spleen, as already described.(16) We found a high positive rate of splenic involvement at ultrasonography by using high-spatial-resolution tissue harmonic compound technology and perfusional study with microbubbles.(16) During the contrast-enhanced ultrasonography parenchymal phase, malignant nodules appeared as clearly circumscribed defects of enhancement, due to the rapid washout of microbubbles as compared with the homogeneously enhanced normal parenchyma. This was likely due to the neoangiogenic vascular structure of the neoplastic lesions, characterized by abnormal endothelial layer with large fenestrations.(16)

*Results at lung sites*

PET/MRI identified in the lung of three patients three lymphomatous nodules with median long axis diameters of 2.5 cm with a range of 2.2-2.5 cm, seated at superior (n= 2) and inferior (n= 1) lobe of the lung. PET/CT identified in the lung of five patients five malignant nodules. The agreement between PET/CT and PET/MRI for number, size and location of pathological nodules in the lung was substantial with k values of 0.733, 0.74 and 0.61 respectively (by Cohen’s test).

The reference standard classified as malignant five nodules in the lung of five patients, who were available for analysis. Compared with the reference standard, there was no significant difference between PET/MRI and PET/CT for sensitivity in detecting pulmonary invasion by lymphoma (60% *vs.* 100%, respectively; *P*= 0.11; by Pearson’s chi-squared test).

*Results at liver sites*

PET/MRI identified in the liver of four patients six lymphomatous nodules: median number of nodules per patient was 1 with a range of 1-3, median of long axis diameter was 2 cm with a range of 1.8-3.0 cm, and involved sites were at right (n= 4) and left (n= 2) lobe of the liver. PET/CT identified in the liver of six patients ten lymphomatous nodules. The agreement between PET/CT and PET/MRI for number, size and location of pathological nodules in the liver was from moderate to substantial with k values of 0.57, 0.73 and 0.53 respectively (by Cohen’s test).

The reference standard classified as malignant ten nodules in the liver of six patients, who were available for analysis. Compared with the reference standard, there was no significant difference between PET/MRI and PET/CT for sensitivity in detecting hepatic invasion by lymphoma (66.6% *vs.* 100%, respectively; *P*= 0.12; by Pearson’s chi-squared test).

*Results at bone sites*

PET/MRI identified in the bone of ten patients 24 lymphomatous lesions: median number of focal lesions per patient was 2 with a range of 1-7, median of long axis diameters was 1.5 cm with a range of 0.5-2.5 cm, and involved sites were at vertebrae (n= 8), femur (n= 5), pelvic bones (n= 3), ribs (n= 3), skull (n= 2), sternum (n= 2) and humerus (n= 1). PET/CT identified in the bone of five patients 14 lymphomatous lesions. The agreement between PET/CT and PET/MRI for number, size and location of pathological lesions in the bone was from moderate to substantial with k values of 0.497%, 0.69%, and 0.69% respectively (by Cohen’s test).

The reference standard classified as malignant 24 lesions in the skeleton of ten patients, who were available for analysis. Compared with the reference standard, there was significant difference between PET/MRI and PET/CT for sensitivity in detecting bone invasion by lymphoma (100% vs. 50%, respectively; *P*< 0.01; by Pearson’s chi-squared test).

Noteworthy, the entire setting of MRI sequences of our protocol was particularly useful for bony sites assessment. In particular, the skeleton lesions were hypointense on T1-weighted Dixon and T2-weighted HASTE images, and presented hyperintensity on STIR, and showed restricted water diffusivity on MRI-ADC maps.

**Supplemental Appendix, 5:**

Statistical analysis was performed using specific software (R version 3.6.0).The generalized kappa statistic (κ) was used to determine inter-modality agreement (PET/CT *vs.* PET/MRI) for occurrence, number, size and location sites of nodal and extra-nodal lymphomatous involvement, and for disease staging, considering: κ<0.2 as slight agreement, κ 0.21 – 0.40 as fair agreement, κ 0.41 – 0.60 as moderate agreement, κ 0.61 – 0.80 as substantial agreement, and κ 0.81 – 1.00 as almost perfect to perfect agreement. Agreement of each imaging modality with standard of reference was also investigated by k statistic. Other statistical evaluations were performed with the Pearson’s chi-squared test.Statistical significance was accepted for *P* values lower than 0.05.

Supplemental Appendix Table 1. Technical details of FDG-PET/diagnostic CT scans

| **CT** | | | **Plane** | **Area scanned** | | **mAs** | **kV** | **Speed (s per rotation)** | | **Thickness** | | **FOV (mm)** |
| --- | --- | --- | --- | --- | --- | --- | --- | --- | --- | --- | --- | --- |
| Attenuation correction | | | Axial | Whole-body | | 80-120 | 80 | 0.94 | | 4.0 | | 700 |
| Diagnostic contrast-enhanced | | | Axial | Whole-body | | 250–380 | 140 | 0.94 | | 4.0 | | 350–500 |
| **FDG** | | | **Injected** | **Start of PET**  **examination**  **from injection** | |  |  |  | |  | |  |
|  | | | Median dose, 360 MBq  Range, 330-400 MBq | Mean time,  71 minutes  SD, ± 15 minutes | |  |  |  | |  | |  |
| **PET** | **BP** | **Acquisition time/BP(min)** | **Iterative reconstruction algorithm** | | **Iterations** | | **Subsets** | | **Axial FOV (mm)** | **Voxel size (mm^3^)** | **Matrix** | |
|  |  | 2 | GE VUE Point FX  With SharpIR | | 3 | | 18 | | 160 | 4 × 4 × 4 | 256 × 256 | |
| **FDG-PET/CT** | **Total time for the study** |  |  | |  | |  | |  |  |  | |
|  | Mean, 18 minutes  SD, ±5 minutes |  |  | |  | |  | |  |  |  | |
| FDG-PET,^18^F-fluoro-deoxy-glucose-positron emission tomography; CT, computed tomography; mAs, milliampere second; kV, kilovolt; FOV, field of view; BP, bed position; GE VUE Point FX with SharpIR, three-dimensional ordered subset expectation maximization (OSEM) with point-spread function (PSF) and time of flight (FOT) technologies; MBq, megabecquerel.  PET data and CT attenuation scans were acquired during shallow free breathing.  PET data underwent automatic attenuation correction using attenuation maps generated from attenuation correction CT.  Diagnostic contrast-enhanced computed tomography scans were acquired during shallow free breathing for head,neck, abdomen, pelvis and upper thighs; they were acquired during breath-hold in expiration for the chest and upper abdomen.  Allpatientsunderwentcontrastinjection. Iodine-based contrast medium Iomeprol (Iopamiro370,BraccoImagingS.p.A.,Milan,Italy) was injected intravenously with a power injector (Empower CTA, Acist Medical Solutions, EdenPrairie,MN,USA) at2mls^—1^, at a fixed dose of 80 ml for patients weighing < 80 kg and at 100 ml for those weighing ≥ 80 kg; scans were started at the end of contrast injection. | | | | | | | | | | | | |

Supplemental Appendix Table 2. Technicaldetails of hybrid FDG-PET/MRI scans

| **MRI**  **sequence** | | **Plane** | | **Area scanned** | **iPat** | **TR**  **(ms)** | **TE**  **(ms)** | **Matrix** | **NEX** | **FOV**  **(mm)** | **Slice Thickness (mm)** | **Gap (mm)** | **FA**  **(degrees)** | **Voxel size (mm)** | **TI**  **(ms)** | **Fat saturation** |
| --- | --- | --- | --- | --- | --- | --- | --- | --- | --- | --- | --- | --- | --- | --- | --- | --- |
| Co-aquired with PET | | | | | | | | | | | | | | | | |
| Dixon T1w 2-Point  VIBE | | Coronal | | Whole-body | 5 | 3.9 | 1st TE 1.23  2nd TE  2.46 | 79×192 | 1 | 500 | 3.1 | 0 | 100 | 2.6×2.6×3.1 |  |  |
| DWI  (b-values 50-800) | | Axial | | Whole-body | 2 | 9700 | 59 | 112x156 | 3b50  6b800 | 420 | 6 | 0.6 |  | 1.7x1.7x6.0 | 220 | Yes |
| T2w HASTE | | Axial and coronal | | Whole-body | 2 | 1400 | 87 | 288x384  (Axial)  256-256  (Coronal) | 1 | 420 | 6 (Axial)  5 (Coronal) | 0.6 | 103 | 1x1x6.0 (Axial)  1.5x1.5x5.0  (Coronal) |  | No |
| STIR | | Coronal | | Whole-body | 3 | 5100 | 104 | 186x384 | 1 | 450 | 5 | 1.5 |  | 1.4x1.4x5.0 | 220-230 |  |
| **PET** | | | | | | | | | | | | | | | | |
| **PET** | **BP** | | **Acquisition time/BP (min)** | | | **Iterative reconstruction algorithm** | | | **Iterations** | | **Subsets** | | **FOV axial (mm)** | **Voxel size (mm3)** | | **Matrix** |
|  | 5 | | 7 | | | AW OSEM 3D | | | 3 | | 21 | | 258 | 2.3 × 2.3 × 5.0 | | 172x172 |
| **PET** |  | | **Start of examination from FDG injection** | | |  | | |  | |  | |  |  | |  |
|  |  | | Mean time, 126 minutes  SD, ± 26 minutes | | |  | | |  | |  | |  |  | |  |
| **FDG-PET/MRI** |  | | **Total time for the study** | | |  | | |  | |  | |  |  | |  |
|  |  | | Mean, 60.07 minutes  SD, ± 25.45 minutes | | |  | | |  | |  | |  |  | |  |
| FDG-PET, ^18^F-fluoro-dexossy-glucose-positron emission tomography; MRI, magnetic resonance imaging; iPat, integrated parallel acquisition technique; TR, time of repetition; TE, time of echo; NEX, number of excitations; FOV, field of view; GAP, slice thickness; FA, flip angle; TI, time of inversion; DWI, diffusion weighted imaging; HASTE, Half Fourier Acquisition Single Shot Turbo Spin Echo T2-weighted; STIR, Short tau inversion recovery; VIBE, Volumetric interpolated breath-hold examination; BP, bed position; AW OSEM 3D, three-dimensional attenuation weighted ordered subsets expectation maximisation iterative reconstruction algorithm; Sd, standard deviation | | | | | | | | | | | | | | | | |
